# Supplementary material for: MetaRibo-Seq measures translation in microbiomes
Source: Nat Commun. 2020 Jun 29;11:3268. doi: 10.1038/s41467-020-17081-z (PMC7324362; doi:10.1038/s41467-020-17081-z)
Supplement: Supplementary file 10 — Supplementary Data 7 [file 41467_2020_17081_MOESM10_ESM.zip › File2/Confidence_VeryHigh_Taxonomy/159225_out.krona.html]

Javascript must be enabled to view this page.

members
magnitude
magnitudeUnassigned
count
unassigned
taxon
rank

159225\_out

5

superkingdom
2
4

1
phylum
976

class
200643
1

order
171549
1

171552
family
1

genus
577309
1

species
454154
1

SRS024140\_contig\_number\_contig-100\_277.27499

phylum
1239
3

3
186801
class

3
186802
order

family
31979
2

1485
genus
2

2

SRS015431\_contig\_number\_63297SRS024132\_contig\_number\_50606
species
1896992


SRS017191\_contig\_number\_19754
1
1898207
species


SRS019068\_contig\_number\_contig-100\_2045.414247
1
